# Advances in NIR-Responsive Natural Macromolecular Hydrogel Assembly Drugs for Cancer Treatment

**DOI:** 10.3390/pharmaceutics15122729

**Published:** 2023-12-04

**Authors:** Chenyu Zhao, Boyue Pan, Tianlin Wang, Huazhe Yang, David Vance, Xiaojia Li, Haiyang Zhao, Xinru Hu, Tianchang Yang, Zihao Chen, Liang Hao, Ting Liu, Yang Wang

**Affiliations:** 1 China Medical University—The Queen’s University Belfast Joint College, China Medical University, Shenyang 110122, China; czhao04@qub.ac.uk (C.Z.); bpan01@qub.ac.uk (B.P.); d.vance@qub.ac.uk (D.V.); 40389982@ads.qub.ac.uk (T.Y.); 18636147887@163.com (Z.C.); 2 Department of Chemistry, School of Forensic Medicine, China Medical University, Shenyang 110122, China; lhao@cmu.edu.cn; 3 Liaoning Province Key Laboratory of Forensic Bio-Evidence Sciences, Shenyang 110122, China; 4 Center of Forensic Investigation, China Medical University, Shenyang 110122, China; 5 Department of Biophysics, School of Intelligent Medicine, China Medical University, Shenyang 110122, China; tlwang@cmu.edu.cn (T.W.); hzyang@cmu.edu.cn (H.Y.); 6 School of Pharmacy, Queen’s University Belfast, Belfast BT7 1NN, UK; 7 Teaching Center for Basic Medical Experiment, China Medical University, Shenyang 110122, China; xiaojia9784@126.com (X.L.); 18909833905@163.com (H.Z.); 8 The 1st Clinical Department, China Medical University, Shenyang 110122, China; stu13324008112@163.com

**Keywords:** near-infrared light, natural macromolecular, hydrogel assembly drugs, cancer treatment, controlled release

## Abstract

Cancer is a serious disease with an abnormal proliferation of organ tissues; it is characterized by malignant infiltration and growth that affects human life. Traditional cancer therapies such as resection, radiotherapy and chemotherapy have a low cure rate and often cause irreversible damage to the body. In recent years, since the traditional treatment of cancer is still very far from perfect, researchers have begun to focus on non-invasive near-infrared (NIR)-responsive natural macromolecular hydrogel assembly drugs (NIR-NMHADs). Due to their unique biocompatibility and extremely high drug encapsulation, coupling with the spatiotemporal controllability of NIR, synergistic photothermal therapy (PTT), photothermal therapy (PDT), chemotherapy (CT) and immunotherapy (IT) has created excellent effects and good prospects for cancer treatment. In addition, some emerging bioengineering technologies can also improve the effectiveness of drug delivery systems. This review will discuss the properties of NIR light, the NIR-functional hydrogels commonly used in current research, the cancer therapy corresponding to the materials encapsulated in them and the bioengineering technology that can assist drug delivery systems. The review provides a constructive reference for the optimization of NIR-NMHAD experimental ideas and its application to human body.

## 1. Introduction

### 1.1. The Hazards of Cancer and the Limitations of Traditional Therapy

Cancer, also referred to as malignant tumor, is a malignant disease caused by malignant cell proliferation. It has the biological characteristics of uncontrolled growth, invasion and metastasis. The treatment of cancer is still an important research topic in the field of global medicine and health. According to the data, in 2022 about 287,850 women in the United States were diagnosed with breast cancer and 43,250 women died of breast cancer [1]. A total of 7,794,799 cases of liver cancer were reported globally in 2022, including 1,447,470 cases in China and 2,014,709 cases in South Asia [2]. In 2023, the United States is expected to have 1,958,310 new cancer cases and 609,820 cancer deaths [3,4]. However, traditional therapies such as surgical resection, radiotherapy and chemotherapy still have problems such as a difficulty in completely removing cancer cells and causing damage to the other tissues of patients [5,6,7,8]. In recent years, with the development of precision medicine and targeted drugs, immunotherapy (IT) has become the mainstream research direction [9]. However, due to individual differences, IT also faces the problems of a low therapeutic effect and severe immunotoxicity [10]. Biodegradable polymers have good biocompatibility and biodegradability compared with other materials. After degradation in vivo, they can be metabolized by the body without any effect. Natural macromolecular hydrogels are easy to synthesize, have low self-toxicity and can be used to selectively deliver drugs, combine different therapies and act as an effective system to treat cancer [11].

### 1.2. Features and Mechanism of Hydrogels for Drug Delivery

Hydrogels are three-dimensional networks formed by chemical or physical cross-linking of hydrophilic polymer chains [12]. When exposed to water, they swiftly expand; they also possess an exceptional ability to retain moisture. Their malleable texture, modifiable form and elevated water content endow them with physical attributes resembling biological tissues, thus bestowing hydrogels with remarkable biocompatibility [13,14]. A hydrogel has a long half-life and high bioavailability in biological organisms [15]. As an effective drug delivery system [16], hydrogels can be used in oncology, cardiology, burn treatment and other medical fields. The cross-linked hydrogel network protects the drugs encapsulated in it, especially bioactive macromolecules that are not subject to inwardly diffusing enzymatic degradation and so this reduces the risk of drug denaturation and aggregation when exposed to organic solvents [17]. The size, structure and design function are factors that together determine how hydrogels will be used for drug delivery [18]. Hydrogels are produced through technology and can generally be made into any shape and size, from being used as from macroscopic hydrogels for wound dressings to nanogels suitable for delivering nucleotide drugs; the size of the hydrogel will determine where the drug is delivered and how the hydrogel will be delivered and how it enters the human body [19]. Judging from its microstructure, cross-linked hydrophilic polymer chains form a grid structure, and the size of the grid will affect the fusion and release of drugs in the hydrogel network [20]. At the molecular and atomic levels, polymer chains can design drug binding sites based on their physical and chemical properties and different polymer chains have different cross-linking methods. The affinity to form coordination bonds and the binding force will macroscopically affect the degradation ability, hardness and other properties of the hydrogel [21]. Whether there are natural coordination bonds in the chain and whether additional cross-linking agents need to be added during the design stage will affect the biocompatibility performance of the hydrogel. The design of the hydrogel drug delivery system must ensure the biological activity of the drug and remain stable during packaging, transportation, storage, etc. For clinical treatment effects, it is necessary to maximize the overall treatment effect and patient compliance. How to release the drugs and regulate them safely and effectively is particularly important. 

Usually, drugs can be released from hydrogels through two methods: degradation and swelling [22,23]. When the drug size is larger than or close to the grid size, the mesh will produce a certain steric hindrance to the drug, which plays a role in sustained release to a certain extent. Increasing the polymer concentration and the cross-linking agent concentration will also shrink the mesh and provide additional friction resistance [24]. As the mesh degrades, the size of the mesh gradually increases, allowing the drug to be released [25]. The rate of mesh degradation can be adjusted according to different degradation and erosion mechanisms, with degradation times ranging from days to months to meet different clinical drug delivery needs [26]. The degradation products in the body should also be harmless and be cleared with human metabolism. The second method releases drugs via swelling [27]. This happens through changes in the balance between the forces that limit mesh degeneration and the osmotic forces that lead to water absorption. As the hydrogel swells, the mesh becomes larger. Drugs are released from hydrogels, and the swelling behavior can respond to the external environment. Therefore, hydrogels with different response types can be designed, such as light [28], pH, glucose and ionic strength responses [29]. In recent years, with the development of nanotechnology and the emergence of precision medicine concepts, anti-cancer treatments using hydrogels as drug carriers are gradually emerging. Various response types for hydrogel drug delivery systems are available in the treatment of different cancers. For example, a pH-responsive hydrogel has a small mesh in the acidic conditions of the stomach and the drug is well wrapped. Under neutral conditions, the swelling rate is the highest. Targeted therapy of cancer can be carried out based on this feature, so that the drug can be absorbed in the tumor. Photo responsive hydrogels undergo photothermal conversion when undergoing photo cross-linking to thermally ablate tumors based on drug treatment. Hydrogels utilizing responsive properties can respond to different external stimuli, allowing for a more controllable way to achieve the release of encapsulated drugs.

### 1.3. The Mechanism of NIR-NMHADs

**Figure 1:**
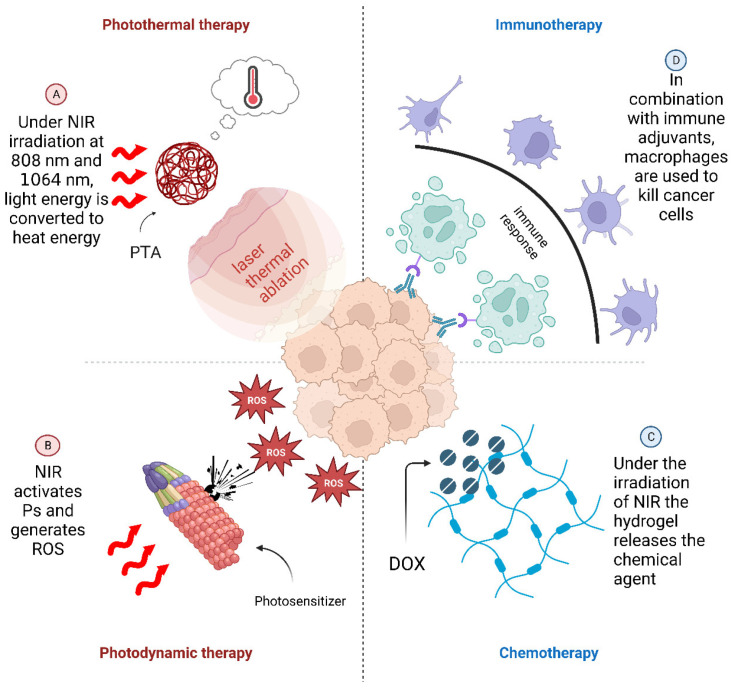
Four anti-cancer therapies derived from the internal wrapping materials of the hydrogel. Reactive oxygen species = ROS. Doxorubicin = DOX.

Researchers have ingeniously engineered hydrogels with stimuli-responsive characteristics, imbuing them with heightened intelligence and adaptability [30]. For example, temperature-responsive hydrogels were prepared by using crown ether derivatives combined with poly (*N*-isopropylacrylamide) and chitosan hydrogels with high pH responses were prepared by using a water-displayed CG model [31]. However, these hydrogels still need a reliable release method during drug delivery. NIR irradiation has become the main drug release method for hydrogels due to its deep penetration of body tissues, minimum cellular damage, space–time controllability and other factors [32]. The treatment process of an NIR-NMHAD is shown in Figure 1. The hydrogel carries the encapsulated drug to the cancerous tissue and then releases the drug under NIR irradiation, thereby achieving a high bioavailability treatment. Meanwhile, natural macromolecular hydrogels have different physical and chemical properties, which in turn affect the overall treatment of drugs. The encapsulated nanomaterials can be roughly divided into four categories: photothermal agents (PTAs), photosensitizers (Pss), chemotherapeutic agents and immune adjuvants. Therefore, the treatment methods of the drugs are also divided into photothermal therapy (PTT), photothermal therapy (PDT), chemotherapy (CT) and immunotherapy (IT) [33,34,35,36]. NIR irradiation has high controllability, so medical staff can control the release of drugs in vitro, and the encapsulated drugs have shown satisfactory results in synergistic therapies [37].

In this review, we highlight the advantages of NIR irradiation and the excellent application prospects of NIR-II (1000–1700 nm) by comparing various commonly used light sources. In addition, we also summarize the commonly used natural macromolecular hydrogels and nanomaterial adjuvants to provide ideas for future experimental design and create better drug delivery systems with bioengineering technology.

## 2. The Application of NIR Light to HAD: Properties, Advantages and Controlled Release

### 2.1. The NIR Light and Properties with Different Wavelength Windows

Near-infrared rays are often used in clinical diagnosis and imaging techniques [38]. The NIR spectral window has the following advantages: (1) Its autofluorescence is very low, which can greatly reduce the background interference of optical imaging technology and is very suitable for cancer-related detection and treatment [39,40,41]. (2) The high tissue permeability and extremely fast propagation speed can ensure the real-time control of drug release in deeper tissues, which is widely used. (3) In the therapeutic application of NIR irradiation for human body, compared with other types of lasers (UV and X-ray), NIR light can almost guarantee being non-invasive and harmless to the body [42]. Because of these excellent properties, researchers have begun to apply NIR technology to the design of new anti-cancer drug delivery systems in recent years [39,43,44,45]. NIR light is sensitive to changes in the shape of lipids or water bands in the human body, so most of the research based on NIR light is focused on cancer detection [46]. Although the ability of near-infrared spectroscopy to analyze the deep tissues of the body is very limited, it is the most cost-effective optical tool for microvascular horizontal tissues [47]. Combined with the high biological affinity of hydrogels, NIR hydrogel drugs are more promising than traditional cancer treatment methods. According to the wavelength range, NIR light is divided into two windows: NIR-I (700–950 nm) and NIR-II (1000–1700 nm) [39]. At present, the application wavelengths of NIR-I and NIR-II are 808 nm and 1064 nm. The wavelength of the NIR light is very important and affects the material selection for the PTA. NIR-I has a low wavelength but high energy, which is suitable for most PTAs and has a wider selection range. In the experiment designed by Luo et al., polydopamine (PDA), whose photothermal efficiency was greater than or equal to 40%, was introduced as a photothermal material that perfectly converts the 808 nm NIR laser energy continuously irradiated for 2 min into heat. Originally, the temperature only increased from 37 °C to 39 °C; after optimization (by adding polydopamine), the temperature could reach 42 °C, which improved the permeability of the cell membrane and enhanced the subsequent release of cisplatin. The experimental design shows that the NIR-I window can effectively inhibit the growth of tumors in vivo [48]. However, NIR-II has stronger biological safety but there are still few PTAs that have been developed. It is difficult to develop single and mixed materials with high photothermal conversion efficiency, so this needs further in-depth study [49]. Ruan et al. chose NIR-II as the light source because the NIR-II window has the advantage of better laser maximum allowable exposure (MPE). They tested the temperature increment for 4-nitrophenyl-β-D-glucopyranoside-polyethylene glycol (PNPG-PEG) composites (25.2 °C and 26.4 °C) and the synthesis temperature of hydrogels (52.3 °C and 53.9 °C) under 808 nm and 1064 nm irradiation, respectively. In addition, they also used chicken breasts with different thicknesses as tissue models and found that the 1064 nm laser temperature change rate was more stable with a change in irradiation depth [50].

With the progress of the material synthesis process, researchers could gradually synthesize encapsulated drugs with optical properties in type II windows. Recently, many experiments have shown this trend. Bin Li et al. synthesized a self-assembly aggregation-induced emission nanosphere. Driven by high-performance NIR-II, the photothermal reaction promotes the decomposition of ammonium bicarbonate and produces many mixed bubbles of carbon dioxide and ammonia, indicating excellent pharmacological properties [51]. Xiaonan Zhang et al. believed that the NIR-I biological window could not cooperate well with conventional chemotherapy scaffolds to achieve on-demand drug release due to its poor internal tissue penetration depth. Therefore, they combined SrCuSi_4_O_10_ and β-tricalcium phosphate to induce the transition of gelatin from gel to sol under photothermal conditions and then triggered the release of DOX in gelatin to kill cancer cells and repair bone defects caused by tumors [52]. In addition, nanozymes with intrinsic enzyme mimic activity have shown the potential to replace natural enzymes in many fields due to their high catalytic stability and easy functionalization [53]. Tao Jia et al. reported a bimodal AgPd plasmonic blackbody nanozyme with a photothermal conversion rate of 45.1% under 1064 nm infrared irradiation. Compared with the untreated control group, the synergistic treatment of nanozyme and PTT reduced the tumor volume by a factor of seven [54].

**Figure 2:**
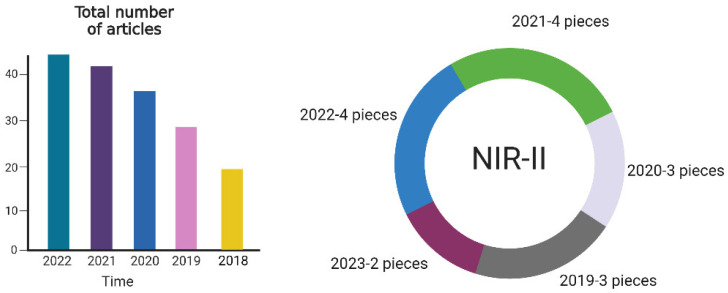
The number of NIR-HAD-related reports in recent years and the prospect analysis of NIR type II light.

Through a web of science search of nearly 5 years of reports, we found that a total of 244 results were obtained using “NIR”, “cancer” and “hydrogel” as keywords. A total of 16 results were found using the NIR-II window for experiments; the earliest year for a report was 2019 (Figure 2). Therefore, using NIR-II as a light-driven source is a novel and promising design idea.

### 2.2. Laser Driving Forces in NIR vs. UV and X-ray Irradition

A laser beam is a high-density beam that is always used to target designated cells and tissues for medical research. In medicine, lasers can be used in a wide range of fields for things such as eliminating cancer cells, removing skin scars and improving vision. In the process of drug action, the commonly used laser driving forces are NIR, UV and X-ray irradiation. UV irradiation has high phototoxicity and low permeability. It often causes irreversible damage to the body when the body is irradiated and has obvious shortcomings in the treatment of deeper tissues. UV irradiation on the skin will cause DNA damage, causing mutations in specific genes and even skin cancer. In light-controlled hydrogel drugs, the response mechanism is in vitro laser irradiation, therefore UV irradiation is not suitable as a light driving force [55]. In addition to being an application light source, UV irradiation can assist in optimizing the overall drug delivery system. In the studies by Chen et al. and Fathi et al., the authors used UV-vis spectrophotometers to determine the release of DOX at 480 nm in order to study the delivery effect of DOX [56,57]. Similarly, X-rays have been an important factor in increasing cancer risk. Excessive X-ray irradiation can lead to constipation, diarrhea and bleeding [49,58]. In contrast, NIR irradiation has unparalleled spatial and temporal controllability, which allows noninvasive and excellent pharmacokinetic characteristics in the therapeutic mechanism. Therefore, NIR has become the laser driving force with the most potential. The table below (Table 1) shows a comparison of the properties of NIR, UV and X-ray irradiation.

### 2.3. NIR-Controlled Hydrogel Release Drugs

**Figure 3:**
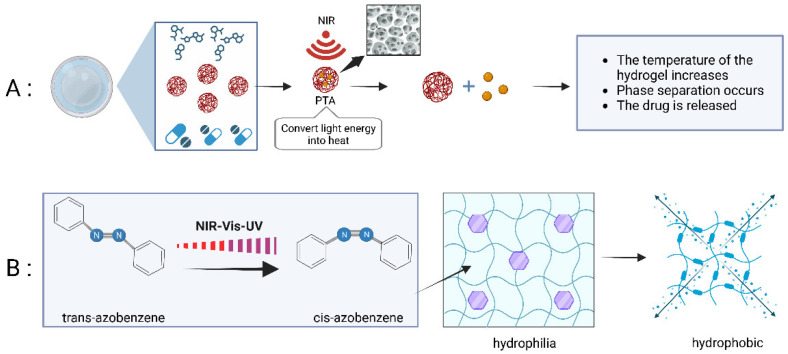
(**A**) The release and control mechanisms of LCST hydrogel and photosensitive hydrogel. (**B**) The spatial structure of azobenzene affects the hydrophilic and hydrophobic properties of hydrogels through their polarity.

The NIR-irradiation-mediated release of nanomaterials is divided into three categories, photothermal reactions, photochemical reactions and photooxidation reactions [62]. In NIR-responsive hydrogels, the photothermal reaction is the most common application. Through the NIR irradiation, the photothermal material absorbs the energy of the laser and converts it into heat energy. Through the regulation of temperature, the hydrogel is transformed between the sol and gel phases to complete the release of the drug. In contrast, the other two reaction types are rare. There are relatively few experiments on the design of photochemical reactions. The mechanism is that the materials with near-infrared absorption capacity, such as coumarin and *o*-nitrobenzyl, change from the ground state to the excited state after absorbing a certain number of photons and undergo rearrangement reactions and photo-cleavage reactions. After destruction, the drug release is completed [63]. Similarly, upconversion luminescence nanoparticle (UCNP) material can convert NIR light into UV light and change azobenzene from a cis-form to a heterogeneous form that can also complete the release of drugs [64] (Figure 3B). The photooxidation reaction happens by positioning Ps at the polymer skeleton; the polymer is then cracked under NIR irradiation to release the assembled drug, but there is little in the current experimental design.

#### 2.3.1. The Thermosensitive Hydrogel Releases Drugs via NIR Irradiation

Temperature-sensitive hydrogels come in two types: thermal expansion ones and thermal shrinkage ones. The swelling degree of the thermal expansion hydrogel increases with an increase in temperature and the macromolecular chain expands rapidly due to hydration at high temperatures. At higher temperatures, the macromolecular chains of the heat-shrinkable hydrogels shrink due to aggregation and the swelling degree also decreases sharply; however, swelling occurs at lower temperatures. At present, most of the research is based on lower critical solution temperature (LCST) materials and there are few designs based on upper critical solution temperature (UCST) materials [65]. The main mechanism for the temperature-sensitive hydrogel to release the drug is that its internal encapsulated material has the property of photothermal conversion. Under NIR irradiation, the PTA converts the NIR light energy into heat energy to achieve the temperature rise in the temperature-sensitive hydrogel (Figure 3A). After reaching the dissolution temperature of the phase separation, the hydrogel will shrink and extrude the internal encapsulated drug. Lima-Sousa et al. prepared injectable in situ-formed thermal reaction hydrogels using agarose and chitosan bound with graphene oxide (GO) and reduced graphene oxide (rGO). The thermal conversion properties of the combined hydrogel drug mainly come from the agarose hydrogel. The researchers tested the swelling rate of thermosensitive hydrogel–GO and thermosensitive hydrogel–rGO. All formulations reached the maximum swelling after incubation for 45 min, with an average of 24%. After 808 nm, 1.7 W/cm^2^ near-infrared irradiation, the survival rate of MCF-7 cancer cells decreased to 34%, reflecting the excellent ability of near-infrared light to regulate drug release [66]. In addition, the hyaluronic acid–polydopamine/doxorubicin nanoparticles (HA-PDA @ IQ/DOX NPs) prepared by Zhang et al. showed a photothermal conversion efficiency of up to 41.2%, which not only achieved full drug release but also accelerated the effect of DOX [67]. The intelligent hydrogel melanin guanosine monophosphate Mel/G/DH designed by Wu et al. showed photothermal activity after NIR irradiation for 5 min and its temperature increased from 26 °C to 46.7 ± 0.5 °C. The hydrogel combined with NIR irradiation activates dendritic cells and initiates the immune response to prevent tumor recurrence [68].

#### 2.3.2. Hydrogels Release Drugs through Photochemical Reactions under NIR Irradiation

**Figure 4:**
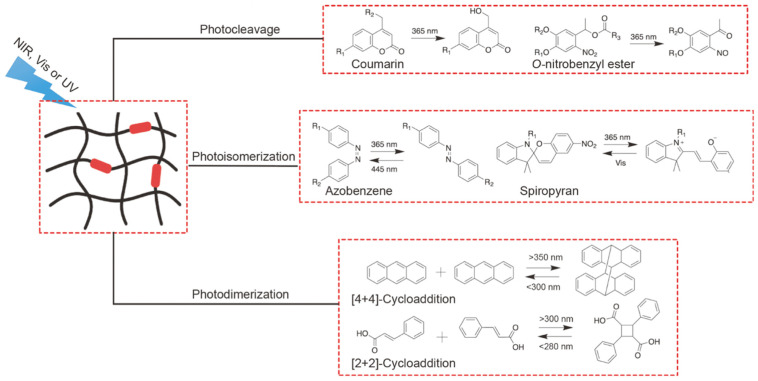
Different representative photoreaction types and photosensitive groups for the design of photosensitive hydrogels.

The commonly used photoreaction of chromophores activated via two-photon absorption (TPA) reaction materials are diazonaphthoquinone, coumarin and *o*-nitrobenzyl (Figure 4). Gulfam et al. developed an NIR-responsive hydrogel extracted from humic acid (HA) and a novel coumarin-based water-soluble cross-linking agent. Compared with the previous photolytic coumarin hydrogel, this design has excellent biocompatibility. They functionalized and formed an inverse electron demand Diels–Alder reaction (IEDDA) by introducing PEG spacers between the two coumarin molecules to produce a new hydrogel whose coumarin–ester bond can be cleaved remotely by near infrared irradiation [69]. However, the small number of chromophores has always been a major disadvantage of this release route. In addition, the smart hydrogel formed by Au nanoclusters and UCNPs exhibits an excellent PTT and PDT synergistic effect. A DNA–UCNP–Au hydrogel was prepared via the electrostatic complexation of salmon sperm DNA (2000 bp) with cationic UCNP–Au NPs. After six cycles of near-infrared (808 nm laser, 1.0 W cm^−2^) irradiation the DNA–UCNP–Au hydrogel exhibited a stable drug release ability [70]. Han et al. designed a hydrogel based on UCNP combined with the mSiO_2_ drug delivery platform and used NIR irradiation to trigger a hydrophobic–hydrophilic switch. Under 980 nm near-infrared light, hydrophobic 2-diazo-1,2-naphthoquinone (DNQ) was converted into hydrophilic 3-indenecarboxylic acid (ICA) to achieve on-demand drug release [71].

## 3. Selection and Application of NIR Functional Hydrogel Materials

According to different their response types, hydrogels can be divided into pH-sensitive hydrogels, electrosensitive hydrogels, thermosensitive hydrogels and light-responsive hydrogels. NIR-HADs have the advantages of low toxicity and controllability for the treatment of cancer [13]. The commonly used NIR functional hydrogel materials include agarose, chitosan, DNA hydrogel and gelatin methacryloyl (GelMA). In hydrogel selection, the following conditions should be considered: (1) Good drug transport capacity. Under the encapsulation of the hydrogel, the nanocarrier is carried to the vicinity of the tumor cells, which is the main reason for the high bioavailability of the HAD. Compared with traditional cancer treatment methods and other emerging therapies, the outstanding advantage of hydrogels is that they can be metabolized by the human body and reduce the residual-drug-mediated damage to the body tissues. (2) Size selection. Hydrogels can also be divided into large gels and microgels. Large gels have slow fluidity and can stay at the reaction site for a long time to increase the efficiency of the treatment. Microgels can carry more nanomaterials to enhance the diversity of the treatment methods [72,73]. In the related experimental design of NIR-HADs, the hydrogel completes the release of the drug under the influence of the material and the near-infrared ray mainly plays the role of activating the internal wrapping material; therefore, the applied hydrogel matrix should be more considered to adapt to the specific material. Here, we will summarize several commonly used NIR functional hydrogel matrices and their latest applications, as well as some innovative hydrogel materials and combinations designed in the laboratory.

### 3.1. Agarose Hydrogel

Agarose is an FDA-approved water-soluble polysaccharide derived from seaweed. It has a low melting point and exhibits reversible melting and gelation behavior. When the temperature is higher than the melting point, the agarose hydrogel begins to release the materials it carries. When the temperature is lower than the melting point, the agarose hydrogel regulates and stops the release of the drug [74]. The controllable release system has high inclusiveness, so the combination of an MXene photothermal agent and an agarose hydrogel is very efficient [75]. HA hydrogel has the advantages of low cost and high biocompatibility; Additionally, its isolate, sodium humate (SH), has high photothermal conversion ability. Therefore, under NIR irradiation, SH is also a new method for treating cancer with agarose hydrogel [76]. In addition, the agarose hydrogel system containing PB is also a safe combination approved by the FDA and shows excellent PTT efficacy [77].

### 3.2. Chitosan Hydrogel

Chitosan is a natural polysaccharide derived from chitin, which exists in arthropod exoskeleton and fungal cell walls. In addition to excellent biocompatibility and biodegradability, the porosity and swelling properties of chitosan hydrogels are key factors for their potential biomedical applications. Porosity is a property of hydrogels that allows nutrients and oxygen to diffuse into the middle of the tissue. Experimental designers can also control the swelling ratio of chitosan hydrogels by changing the cross-linking density, concentration or degree of deacetylation of chitosan, thereby enhancing the superiority of chitosan over other biomaterials [78]. NIR irradiation is a good light-driven source that can transfer energy to the chitosan hydrogel and make the latter perform well in terms of its swelling properties. Because chitosan can be gelated in the presence of NaHCO_3_ in an experiment, Sabino et al. designed an injectable in situ-formed ionic cross-linked hydrogel based on chitosan that showed great potential in the chemo-PTT of breast cancer cells [79]. In another chemo-PTT for colon cancer, with the assistance of β-glycerol phosphate disodium salt (β-GP), the hydrogel-assembled drug tightly encapsulated the PTA and DOX in the body, avoiding the loss of the drug. At the same time, the temperature change during the photothermal conversion process ensured the continuous release of the drug and realized the efficient treatment of bacteria and tumors [80]. Recently, composite hydrogels have also become one of the directions for experimental personnel to select materials, such as the combination of agarose and chitosan. Both have excellent biocompatibility and biodegradability. In addition to being able to load anticancer nanomaterials, they can also be used for wound dressings and regenerative medicine [81]. Injectable self-healing polysaccharide hydrogel is a drug carrier pursued by researchers due to its minimally invasive and local autonomous healing ability [82]. In one study, Zhiyi Qian and his colleagues designed a self-healing polysaccharide hydrogel formed by the dynamic imine bond between aldehyde-modified methylcellulose (MC-CHO) and carboxymethyl chitosan (CMC) through the aldehyde group and the amino group to achieve efficient synergistic therapy safely and stably [83].

### 3.3. GelMA Hydrogel

GelMA is a double-bond-modified gelatin with a water-soluble photoinitiator that can be cross-linked and cured into hydrogels with tunable mechanical properties [84]. This reaction can be carried out under mild conditions and can be controlled in time and space [85], which provides new ideas for the study of biological cell–material interactions [86]. Gelatin itself contains arginine-Gly-aspartic acid sequences [87] and matrix metalloproteinase (MMP) [88] and other target sequences that are similar to natural extracellular matrix hydrogels [86], giving GelMA the characteristics of both natural and synthetic biomaterials. GelMA has excellent biocompatibility and cell reactivity characteristics and can be used to replace artificial basement membranes [89] and other natural collagen hydrogels in the field of tissue engineering [90]. GelMA hydrogels also have a porous 3D structure that can be used in the design of drug delivery systems. GelMA hydrogels also have diverse adjustable pore sizes. Generally, by changing the concentration of GelMA and the photoinitiator [91], other materials can also be added to change the size of the pore; additionally, the size of the pore can be changed by changing the concentration of methacrylic anhydride, the cooling rate or temperature gradient [92]. Taotao Liu et al. also focused on the effect of the freezing temperature and freezing time of the GelMA prepolymer on pore size [93]. GelMA hydrogel as a carrier carrying appropriate materials can be used in the treatment of cancer with different therapies [94], and synergetic irradiation with NIR can also enhance the corresponding effect [95].

### 3.4. DNA Hydrogel

In recent years, a DNA smart hydrogel that combines a DNA biological activity and a hydrogel assembly function has rapidly expanded into the biomedical field [96]. DNA is an important biological macromolecule with the function of carrying and storing genetic information. Because DNA is modifiable, structural modifications can be made for specific diseases. A DNA hydrogel can achieve specific treatment by changing its swelling rate and mechanical properties [97]. NIR light has certain safety and will not damage the internal composition of the DNA hydrogel [98]. The advantages of rolling circle amplification (RCA)-based DNA as a biomaterial are not only that it is biodegradable and biocompatible but also that it can stimulate an immune response and transfer immune adjuvant CpG and photosensitive components. In addition, the use of chemical cross-linking agents can be avoided when designing DNA hydrogels and there is no need to worry about the harm caused to the body by chemical by-products. The treatment results for colon cancer CT26 are also objective. Therefore, DNA hydrogels may be good candidates for the construction of drugs designed to treat cancer with the immune response [68].

In addition to the above commonly used hydrogel matrices, there are also many new synthetic hydrogel materials for NIR-HADs. We summarize the other new synthetic hydrogel materials for NIR-HADs in the below table (Table 2).

## 4. Hydrogel-Based Phototherapy Combined with Other Therapies

### 4.1. Hydrogel-Based Phototherapy Combined with Other Therapies

Phototherapy generally refers to photodynamic therapy and photothermal therapy. The combination of photodynamic therapy and photothermal therapy is an effective way to improve the therapeutic effect of tumor phototherapy. Photothermal conversion can improve the transportation of photosensitizers within tumor cells and enhance the efficacy of photodynamic therapy. Although the combination of traditional phototherapy methods can have a good effect on tumor treatment, its phototoxicity limits its further clinical promotion. The main reason for phototoxicity is that the distribution of phototherapy drugs is not controllable, resulting in reduced accumulation in tumors and weakened efficacy [104]. Phototherapy drugs flowing into other parts of the body will cause damage to healthy cells. Therefore, a method that can carry out phototherapy is needed, as is a drug delivery system that stabilizes and sustains the release of drugs to tumors. Due to their characteristics, hydrogels can achieve the local delivery of phototherapy drugs and at the same time reduce the distribution of phototherapy drugs to non-target sites, reducing phototoxicity while enhancing the therapeutic effects. In addition, hydrogels also provide an opportunity to combine different therapies (such as chemotherapy and immunotherapy) to jointly demonstrate excellent synergistic anti-tumor effects [105]. For example, Shu Zhu and colleagues used a hydrogel as a carrier to carry indocyanine green and Cu-hemin. Under the irradiation of NIR-II light, indocyanine green acted as a photothermal agent and a photodynamic agent at the same time, converting light energy into heat energy. Because of this, the hydrogel matrix softens and Cu-hemin is released. Subsequently, the overexpressed GSH in the TME is down-regulated by Cu-hemin, thus amplifying the ICG-mediated PDT. This work combines light therapy, immunotherapy, and chemotherapy. The therapies promote each other and use the hydrogel as a platform to jointly enhance the therapeutic effect on tumors [106].

### 4.2. Photothermal Therapy (PTT) for Cancer Treatment

**Figure 5:**
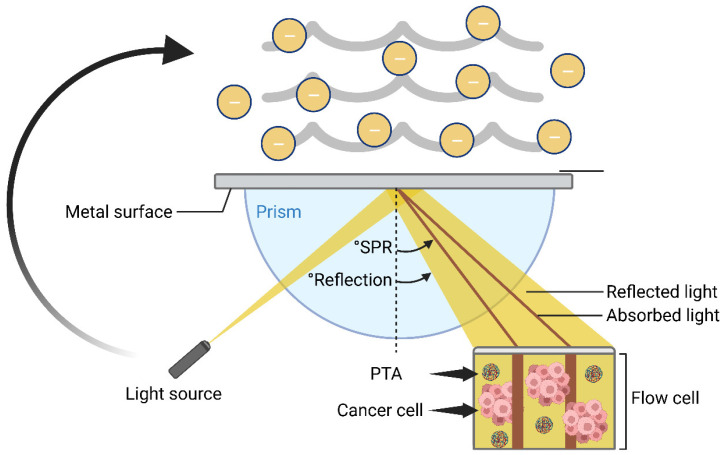
The mechanism of light to heat conversion by surface plasmon resonance (SPR). When the incident light resonates with the vibration of the free electron gas on the surface of the metal nanostructure, the surface plasmon resonance is formed, resulting in local heat.

PTT is a minimally invasive tumor treatment method with the advantages of high efficiency [104], strong controllability and limited side effects [105]. The incident light resonates with the vibration of the free electron gas on the surface of the metal nanostructure, this forms surface plasmon resonance resulting in local heat [106]; photothermal conversion PTAs are used to convert light energy into heat energy [107] so that the temperature of the tumor area increases and the thermal ablation of tumors is achieved (Figure 5) [108]. PTT can be combined with a hydrogel by using its water absorption properties. The characteristic of strong water retention ability make it a carrier to carry different materials to achieve different therapeutic effects. Hydrogels can also be modified to design more intelligent hydrogels, such as light-responsive hydrogels [109] and pH-responsive hydrogels [110], in order to better control drug release. The PTA is very important in PTT; moreover, the photothermal conversion efficiency greatly affects the therapeutic effect of PTT. Generally, PTAs can be divided into inorganic nanomaterials (nanogold [111], metal sulfide [112], gold sulfide [113], etc.), organic nanomaterials (polyaniline [114], polydopamine [115]) and near-infrared absorption dyes (ICG [116], etc.). Indocyanine green (ICG) is an FDA-approved in vivo application dye with low toxicity and fluorescence characteristics that can target cells. Mxene can be used in bone tissue regeneration. Based on thermal ablation, HTA can also be combined with immune adjuvant to produce an immune response. PDA NPS is widely used in a variety of cancer treatments, and Au has strong photothermal conversion efficiency.

Table 3 lists some examples of PTAs used in the treatment of breast cancer, melanoma and osteosarcoma.

### 4.3. Photodynamic Therapy (PDT) for Cancer Treatment

**Figure 6:**
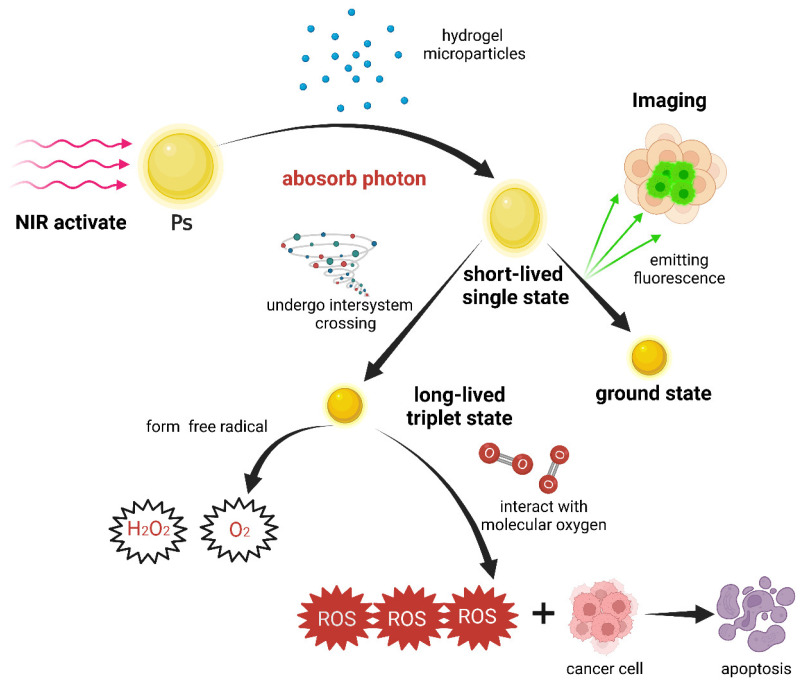
The mechanism of NIR activation of a Ps to achieve PDT.

PDT is a method of using light of a corresponding wavelength to irradiate a Ps so that the Ps can change from a ground state to an excited state (Figure 6). The photosensitive substance in the excited state is extremely unstable and will quickly return to the ground state and release energy. In this process, fluorescence can be generated for further analysis and diagnosis. At the same time, a large amount of ROS are generated [128]. The oxidation reaction occurring in close proximity to biological macromolecules engenders cytotoxicity, leading to the demise of afflicted cells. Such a therapeutic approach holds promise for the treatment of cancer. Traditional therapy can activate Pss that selectively accumulate in the lesion tissue by irradiating the lesion site with visible light, causing photochemical reactions that destroy the lesion. However, traditional therapy has low penetration efficiency for tumors and can only reach a depth of about 1 mm to 1 cm around the lesion site. The new generation of PDTs use NIR light [129], which has higher penetration efficiency, weaker autofluorescence intensity and lower phototoxicity than other types of light. Furthermore, different hydrogels can be used as carriers to carry the appropriated materials to treat cancer in many ways. ICG was encapsulated in Pt (IV) prodrug-initiated hydrogel microparticles (MICG-Pt) prepared by photo-responsive GelMA hydrogel using microfluidic technology. Under irradiation with NIR, the Pt (IV) solidifies the GelMA hydrogel and the large amount of ROS produced by ICG stimulates the Pt (IV) prodrug to generate highly cytotoxic Pt (II) that kills cancer cells. The large amount of ROS produced by ICG can also further generate oxygen and improve the problems caused by hypoxia in the tumor [130].

Table 4 lists some examples of Pss used in cancer treatments.

### 4.4. Hydrogel-Based Phototherapy Combined with Chemotherapy (CT) for Cancer Treatment

Chemotherapy (CT) is a systemic treatment method that uses chemical drugs to prevent cancer cell proliferation, invasion and metastasis until the cells are eventually killed. The commonly used chemotherapy drugs can be divided into tisanes (paclitaxel, albumin-paclitaxel, docetaxel), antibiotics (doxorubicin) and antimetabolites (fluorouracil, methotrexate). The killing of healthy cells by chemotherapy drugs has led to a series of serious systemic side effects in patients. Problems such as the inability to target tumor heterogeneity, low bioavailability and drug resistance have plagued clinical trials. Photothermal therapy can generate heat and enhance the permeability of the extracellular matrix, cell membranes and blood vessels. This physiological change can enhance the concentration of chemotherapy drugs in tumor tissues; at the same time, chemotherapy drugs can enhance the sensitivity of tumor cells to photothermal therapy.

Currently, researchers are seeking more ideal chemotherapy methods, such as using ferroptosis or designing different types of prodrugs to achieve treatment goals, and designing ideal drug delivery systems based on different materials to achieve the release of chemotherapy drugs and reduce the damage of chemotherapy drugs to the human body. Hydrogel-based drug delivery systems, as a platform, can enhance the efficacy and reduce the toxicity of chemotherapy agents while achieving phototherapy to solve the limitations of chemotherapy. Researchers encapsulated doxorubicin in a hyaluronic acid hydrogel, regulating the release of chemotherapy drugs by stimulating hydrogel cross-linking through the ROS generated by ICG [132]. Yuan Cao et al. designed a GO-Fe_3_O_4_ nanomaterial composed of Go embedded in an interpenetrating polymer to improve the performance of a poly(n-isopropylacrylamide) (PNIPAM) hydrogel under the action of near-infrared light and alternating magnetic fields. The solution temperature accelerates the thermal motion of the molecules and promotes the release of DOX. In addition, the addition of alginate makes the hydrogel pH responsive, so a hydrogel with a triple response to pH, near-infrared light and magnetic fields was designed [133,134]. Hou et al. combined peptides with Ag_2_S quantum dots through engineering technology to create a new photothermal agent; together with DOX and the immune adjuvant drug phenylbutyrate, an injectable nanogel system was constructed. This new system provides phototherapy, chemotherapy and immunotherapy for tumor treatment in an effective combination. Zhang et al. mixed chitosan polymer micelles and pegylated gold nanorods loaded with the anti-tumor drug cyclophosphamide (PTX) into a thermosensitive hydrogel to achieve precise tumor targeting during phototherapy and efficient treatment [135]. Amin Ghavami Nejad et al. used dopamine nanoparticles (DP) as a photothermal agent and the anticancer drug bortezomib (BTZ) to jointly construct a stimulus-responsive hydrogel. When the composite hydrogel is irradiated, the DP nanoparticles absorb the light, which is emitted locally as heat to affect cancer cells through hyperthermia. On the other hand, the anti-cancer drug BTZ was successfully released from the hydrogel due to dissociation from catechol. This hydrogel system can ensure that the drug is effectively released under the acidic conditions in tumor cells and achieve an effective combination of phototherapy and chemotherapy [136]. Theune et al. used thermosensitive polymers of PNIPAM and incorporated the photothermal agent polypyrene (PPY) in combination with phototherapy and chemotherapy for cancer treatment [137].

### 4.5. Hydrogel-Based Phototherapy Combined with IT for Cancer Treatment

IT refers to a treatment method that artificially enhances or suppresses the body’s immune function according to the body’s low or high immune status to achieve the purpose of treating diseases. There are many methods of IT that are suitable for the treatment of various diseases. Tumor IT aims to activate the human immune system by relying on its own immune function to kill cancer cells and tumor tissues. Unlike previous surgeries, chemotherapy, radiotherapy and targeted therapies, IT does not target tumor cells and tissues but the body’s own immune system. The materials used in IT are in a more multifunctional form. In recent years, synergistic treatment of tumors with phototherapy and immunotherapy has proven to be effective. PDT can effectively induce tumor cells to release danger-associated molecular patterns and tumor-associated antigens (TAAs). TAAs play a crucial role in inducing immune responses; PTT has also been shown to promote immune responses by triggering immunogenic cell death.

**Figure 7:**
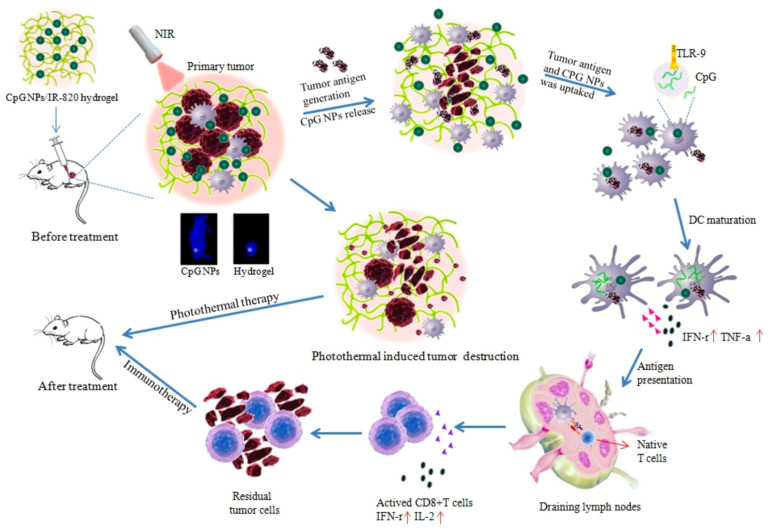
Scheme for CpG NPs/IR820-hydrogels for synergistic photothermal immunotherapy. Reprinted with permission (Fluorescence Imaging Guided CpG Nanoparticles-loaded IR820-hydrogel for Synergistic Photothermal Immunotherapy).

Hydrogels can serve as effective drug delivery systems that enable local administration and effectively synergize phototherapy and immunotherapy for tumor treatment. HTA-containing hydrogels are injected into humans to reprogram tumor-associated macrophages (TAMs) into M1-type macrophages, inducing K7M2 osteosarcoma-associated cell apoptosis and the thermal ablation of tumors [93]. Using M/A-CDs as carriers (CpG ODNs) to induce dendritic cell maturation through mannitol receptors and inducing ICD and DAMPS [117], Ziying Fei et al. designed an injectable hydrogel based on red blood cells. The hydrogel can be activated by physiological signals such as platelets and thrombin. In addition, under irradiation with NIR light, the fragments of TAAs generated after the thermal ablation of tumors can stimulate the body’s immune response, effectively synergizing with phototherapy and immunotherapy [138]. Dong et al. developed a self-assembled injectable hydrogel using new indocyanine green (IR820) as the photothermal agent and combining cytosine–guanine dinucleotide (CPC) nanoparticles with it. Under NIR irradiation, IR820 generates heat, destroys tumor cells and releases antigens. In addition, CPC can also regulate the immune function of a variety of cells, including CD8+ cells, dendritic cells, etc., improving phototherapy and immunity and the effectiveness of synergistic treatments (Figure 7) [139].

## 5. Targeted Drug Delivery Technology for Assisting NIR-HAD Therapy

Spring-type microrobots have high efficiency in high-viscosity liquids such as blood and have excellent targeted cancer treatment capabilities due to their small size. The EMA system can accurately direct the microrobot to the lesion site and improve the bioavailability of the drug. Lee et al. prepared a temperature-responsive hydrogel using perfluoroalkoxy (PFA) microtubules and injection pumps. The magnetic nanoparticles (MNPs) were loaded inside the hydrogel and then the spring-type medical microrobot was electromagnetically manipulated into the specified position, where the loaded therapeutic agent was released by NIR stimulation (heating to 45 °C). In addition to microrobots, the application of nanomotors in pharmaceutics has gradually matured in recent years, achieving high motion efficiency [140,141,142]. Wang et al. developed a 1064 nm NIR-II light-driven hydrogel that combines PDA, DOX and Fe_3_O_4_ @ Cu_9_S_8_ NPs. Fe_3_O_4_ @ Cu_9_S_8_ NPs produced a temperature gradient under irradiation with NIR light, resulting in thermophoresis propulsion and moving the drug to the cancerous area. The asymmetric heat distribution enhances the deep penetration of nanomotors at the tumor site and further enhances the immune response [143]. The microgels generated by the microfluidic device have high surface-to-volume ratio and injection delivery ability. The AuNRs in the hydrogel were heated using NIR light, resulting in the destruction of the hydrogel network and the release of a large amount of drug. In addition, ICG can also be encapsulated into microgel particles by microfluidic technology, and this has effective PTT/PDT performance under NIR light [144,145].

## 6. Conclusions and Perspectives

In this review, the properties and advantages of NIR irradiation, commonly used NIR-functional hydrogel matrices, hydrogel-encapsulated drugs and various treatment methods and commonly used bioengineering-assisted drug delivery systems are systematically summarized.

(1) NIR irradiation has space–time controllability and can well control the instantaneous release of drugs in vivo. The study of the NIR-I window is more mature and has a large number of PTA adaptations. NIR-II has stronger tissue penetration and is more promising for clinical application in the future. Compared with other light sources, NIR is almost non-invasive to the human body and can cooperate with many bioengineering technologies. At present, there are two commonly used NIR-mediated hydrogel drug release methods: one is to convert the light energy of NIR into heat energy through a PTA, change the phase state or swelling rate of the hydrogel and achieve the drug release effect. The other is to convert NIR light into UV light using UCNPs. Then, the hydrogel can be cracked by photochemical reaction to complete the release of the drug.
(2) We introduced the commonly used hydrogel matrices, such as agarose, chitosan, DNA and GelMA hydrogels. In addition, we also list many innovative synthetic hydrogels for reference. The temperature-sensitive, photo-sensitive and pH-sensitive properties of these hydrogels well cater to the application of NIR and establish an efficient and safe drug delivery system.
(3) NIR responsive hydrogel assembly drugs for cancer treatment methods are extremely diverse and the many materials that can be wrapped can cooperate with each other to make up for the shortcomings of a single material, synergistic photothermal therapy, photodynamic therapy, chemotherapy and immunotherapy. Driven by NIR irradiation, effective anti-cancer treatments prevent cancer recurrence.
(4) Bioengineering technology can be roughly divided into two categories in NIR-responsive hydrogel assembly drugs: imaging technology and targeted drug delivery technology. Imaging technology makes the process and results of drug treatment in vivo more intuitive to researchers. Targeted drug delivery technology makes the drug delivery process more controllable and efficient. With the help of these technologies, the design of NIR-responsive hydrogel drugs can be optimized for more applications.

Through this article, researchers can clearly understand the design ideas and application materials of NIR-HADs, and this may help them design better experimental studies with excellent biocompatibility and bioavailability. Hydrogel is a natural and biodegradable drug carrier. NIR is a non-invasive technology that can penetrate into tissues and control drug release in real time. The safety of the combination of the two is the core spirit of the current clinical application. The drugs that can be encapsulated in hydrogels are very diverse and so the derived treatment methods are endless. Bioengineering technology can help to improve the overall bioavailability of the drug and also has a certain optimization prospect. At present, a large number of experimental studies are screening the safest and most efficient experimental design ideas. The experimental design of NIR-HADs is theoretically mature but there are still no human-based experiments. The in vitro release and control drug action points of NIR irradiation for different types of cancer still need to be explored. The wavelength of the NIR light and the drug encapsulated inside the hydrogel are slowly being tested in a large number of experiments to find the optimal adaptation combination; this will take time and the accumulation of a number of experiments. It is believed that, in the near future, NIR-HADs will be used in human experiments, become the main treatment method for cancer and overcome the problems of cancer treatment.

## Figures and Tables

**Table 1:** Comparison of properties among NIR, UV and X-ray irradiation.

	NIR	UV	X-ray	Citation
Wavelength/nm	780–2526	10–400	0.01–10	[59]
Depth of penetration	50–80 mm	The derims can be reached	Whole body	[60]
Harm to human body	Hardly any	Pruritus, dermatitis and skin cancer	Cells will be ionized, affecting cell activity and resulting in cell death	[61]

**Table 2:** Other new synthetic hydrogel materials for NIR-HADs.

Hydrogel	Property	Cancer	Citation
LEFPG	more cell adhesion and proliferation were supported	melanoma	[99]
PNSI	easy to prepare and has good biocompatibility	melanoma	[100]
P407	safe and suitable for ICI;approved by the FDA	colon cancer	[101]
F127	high drug loading capacity and low toxicity	hepatocellular carcinama	[102]
SISMA/ChiMA	suitable for in situ deposition and room-temperature polymerization	melanoma	[103]
OSA/HPCS	injectable and high biocompatibility	breast cancer	[83]

**Table 3:** Some examples of PTAs used in the treatment of breast cancer, melanoma and osteosarcoma.

Photothermal Agent	Cancer	Mechanism of Material Action	Citation
ICG	Breast cancer	The high photothermal efficiency maintains the high concentration of metformin in the tumor, which provides an excellent synergy between PDT and IT	[117]
	Melanoma	Combination of targeted drug therapy and PTT	[118]
MXenes	Osteosarcoma	Thermal ablation of residual tumor cells can effectively promote bone tissue regeneration and has antibacterial properties	[119]
HTA	Osteosarcoma	Induction of apoptosis in osteosarcoma-related cells and thermal ablation of tumors	[120]
SPIIN	Breast cancer	Tumor growth and metastasis to the lung were inhibited by 1064 nm light irradiation	[121]
PDA NPS	Melanoma	Dual regulation of pH and NIR can improve the tumor’s hypoxic environment and maintain drug concentration	[122]
	Breast cancer	Cooperates with PPT and IT	[123]
	Osteosarcoma	High photothermal effect and good biocompatibility provide a good carrier for drug delivery	[124]
DOX/DOPA-rGO	Breast cancer	Combined photothermal and CT effectively reducing the viability to 21%	[125]
Au	Melanoma	Gene-targeted therapy for ocular melanoma under photothermal synergy	[126]
	Breast cancer	The gold coating was attached to enhance the fluorescence signal for easy diagnosis, and the photothermal conversion efficiency reached 65%	[127]

**Table 4:** Some examples of Pss used in cancer treatments.

Photosensitizer Name	Mechanism of Action	Citation
PpIX	ROS is produced to kill tumors, and ROS prodrug release is stimulated to enhance anti-tumor immunity	[131]
	ROS was generated to achieve local PDT treatment, and hydrogel cross-linking was induced to achieve drug release under NIR regulation	[132]
T1-PPa	Two-photon infrared light irradiation improves the depth of light penetration and prevents Ps aggregation	[121]
ICG	PDT in combination with IT for colon cancer; the production of large amounts of ROS and the production of NO inhibit the proliferation of cancer cells	[122]
UZC@HA	Targeted therapy, without damage to normal tissues, induces the apoptosis of breast-cancer-related cells	[123]
